# The effects of regular home delivery of HIV self‐testing and follow‐up counselling on HIV testing and prevention outcomes in men who have sex with men who test infrequently in the United States: a pragmatic, virtual randomized controlled trial

**DOI:** 10.1002/jia2.26318

**Published:** 2024-07-17

**Authors:** Tyler B. Wray, Philip A. Chan, Jeffrey D. Klausner, Lori M. Ward, Erik M. S. Ocean, Daniel J. Carr, John P. Guigayoma, Siddhi Nadkarni

**Affiliations:** ^1^ Department of Behavioral and Social Sciences Brown University School of Public Health Providence Rhode Island USA; ^2^ Department of Medicine Warren Alpert Medical School of Brown University Providence Rhode Island USA; ^3^ Department of Population and Public Health Sciences Keck School of Medicine University of Southern California Los Angeles California USA; ^4^ Department of Population Health Science John D. Bower School of Population Health University of Mississippi Medical Center Jackson Mississippi USA; ^5^ School of Psychology Cardiff University Cardiff UK; ^6^ Yale School of Medicine New Haven Connecticut USA

**Keywords:** HIV self‐testing, gay men, men who have sex with men, pre‐exposure prophylaxis, linkage to care, telehealth

## Abstract

**Introduction:**

Past research shows that HIV self‐testing (HIVST) can increase testing and facilitate more HIV diagnoses relative to clinic testing. However, in the United States, the use of HIVSTs is limited due to concerns that those who use HIVST could be less likely to be linked to care.

**Methods:**

From January 2019 to April 2022, we recruited 811 men who have sex with men (MSM) in the United States who tested infrequently using an online marketing campaign and randomized them 1:1:1 to receive one of the following every 3 months for a year: (1) text message reminders to get tested at a local clinic (control); (2) mailed HIVST kits with access to a free helpline (standard HIVST); and (3) mailed HIVST kits with counselling provided within 24 hours of opening a kit (eTest). Quarterly follow‐up surveys assessed HIV testing, sexually transmitted infection (STI) testing, pre‐exposure prophylaxis (PrEP) use and sexual risk behaviour.

**Findings:**

Eight participants were diagnosed with HIV, and all but one were through HIVST. Participants in either HIVST condition, standard or eTest, had significantly higher odds of any testing (*OR* = 7.9, 95% CI = 4.9−12.9 and *OR =* 6.6, 95% CI = 4.2−10.5) and repeat testing (>1 test; *OR* = 8.5, 95% CI = 5.7−12.6; *OR =* 8.9, 95% CI = 6.1−13.4) over 12 months relative to the control group. Rates of STI testing and PrEP uptake did not differ across study condition, but those in the eTest condition reported 27% fewer sexual risk events across the study period relative to other groups.

**Conclusions:**

HIVST vastly increased testing, encouraged more regular testing among MSM, and identified nearly all new cases, suggesting that HIVST could diagnose HIV acquisition earlier. Providing timely follow‐up counselling after HIVST did not increase rates of STI testing or PrEP use, but some evidence suggested that counselling may have reduced sexual risk behaviour. To encourage more optimal testing, programmes should incorporate HIVST and ship kits directly to recipients at regular intervals.

## 1 INTRODUCTION

Annual rates of new HIV cases in the United States have declined in recent years but are not falling sharply enough to meet Ending the Epidemic goals (EHE) [1, 2], primarily due to underutilization of several effective prevention strategies, including treatment‐as‐prevention and pre‐exposure prophylaxis (PrEP) [2]. Men who have sex with men (MSM) continue to account for the majority of new cases [2, 3]. Testing is the cornerstone of HIV prevention, given that it can facilitate earlier diagnosis and treatment initiation which, in turn, reduces transmission to HIV‐negative partners [4]. Testing could also serve as a key opportunity to link recipients to other prevention methods like PrEP and counselling about ways to reduce risk. For particularly at‐risk populations like MSM, the Centers for Disease Control recommend testing at least every year, but more frequently if they have multiple sex partners [5]. However, few MSM test this frequently [6], suggesting that additional strategies are needed to encourage more regular testing.

HIV self‐testing (HIVST) could be one tool that helps facilitate more frequent testing. One meta‐analysis showed that, across ten studies, HIVST increased testing rates relative to clinic testing, but had a relatively modest aggregate effect size [7]. HIVST also found more new HIV cases than standard of care. Only a handful of past studies trials in high‐income countries have explored the effectiveness of providing multiple HIVST in supporting more regular testing [8, 9, 10, 11]. Instead, most past trials used systems that required participants to order each HIVST themselves [12, 13, 14]. Engaging MSM who are at high risk for HIV and delivering HIVSTs to them at regular intervals could help encourage more regular testing and earlier diagnosis.

Another issue limiting the utilization of HIVST is the fear that those who use HIVST could be less likely to access care when needed, such as testing for other sexually transmitted infections (STIs), PrEP or confirmatory HIV testing and care. A meta‐analysis of four trials of MSM suggested that fewer individuals who used HIVST were linked with care following a reactive test versus standard of care [7]. Fewer studies have tested HIVST's effects on accessing STI testing, and results are mixed [9, 10, 15]. Given these issues, offering active, timely follow‐up counselling alongside HIVST may be one way to capture the benefits of HIVST while also encouraging recipients to seek other services.

In this pragmatic, virtual clinical trial, we conducted online marketing campaigns to enroll MSM who were at high risk for HIV and who tested infrequently and randomized them to receive one of the following every 3 months for a year: (1) text messages reminding them to seek HIV testing at a local clinic (control); (2) HIVST kits mailed to participants’ preferred address with access to a free, 24‐hour helpline (standard HIVST); or (3) mailed HIVST kits equipped with technology that enabled study staff to reach out to participants via phone to offer counselling within 24 hours of opening a kit (eTest). Outcomes were assessed via online surveys collected semi‐quarterly (at 1, 4, 7, 10 and 12 months) over a period of 12 months. Surveys were collected at these staggered intervals relative to test deliveries (baseline, 3, 6 and 9 months) to avoid surveys inadvertently prompting participants to use their tests.

## 2 METHODS

We recruited participants in EHE jurisdictions in the Northeast (Boston), West (Los Angeles) and several areas in the South (Louisiana, Mississippi, Florida; see Appendix A for a full list) from January 2019 to April 2022. We focused on these geographic areas because they provided regional diversity and have high HIV incidence. Procedures were approved by the Brown University Institutional Review Board and were pre‐registered on ClinicalTrials.gov (NCT03654690).

### 2.1 Recruitment

Eligible participants were (1) 18+, (2) self‐reported assigned male at birth, (3) HIV negative or unknown status, and reported (4) not taking or prescribed PrEP for the past 6 months, (5) (a) having engaged in condomless anal sex (CAS) with a partner who was not sexually exclusive, (b) currently having anal sex with an HIV‐positive partner or (c) having been diagnosed with an STI in the last 6 months, and (6) not having tested for HIV in the past 6 months. Eligible participants also (7) reported owning a smartphone, (8) reported having a residence where they could securely receive packages, and (9) resided in one of the eligible locations.

Potential participants were recruited from popular websites and social media platforms. Interested users completed an online survey that assessed basic eligibility criteria. Eligible participants were then asked to provide informed consent and register. As a final step to enrol in the study, participants were informed that research staff would reach out via phone to confirm their personal information. Those who completed this step were considered formally enrolled.

### 2.2 Randomization

Participants were randomized 1:1:1 using a random number generator to receive one of the following every 3 months over the 12‐month study period: (1) text message reminders to get tested for HIV at a local clinic (control); (2) OraSure® OraQuick Advance HIV 1/2 self‐testing kits (OraSure® Technologies, Bethlehem, PA, USA) without follow‐up (standard HIVST); and (3) the same HIVST kits equipped with a sensor (Estimote, Inc., New York City, NY, USA) that alerted staff HIV test counsellors to initiate phone counselling within 24 hours of a test kit being opened (eTest).

### 2.3 Procedures

**Figure 1:**
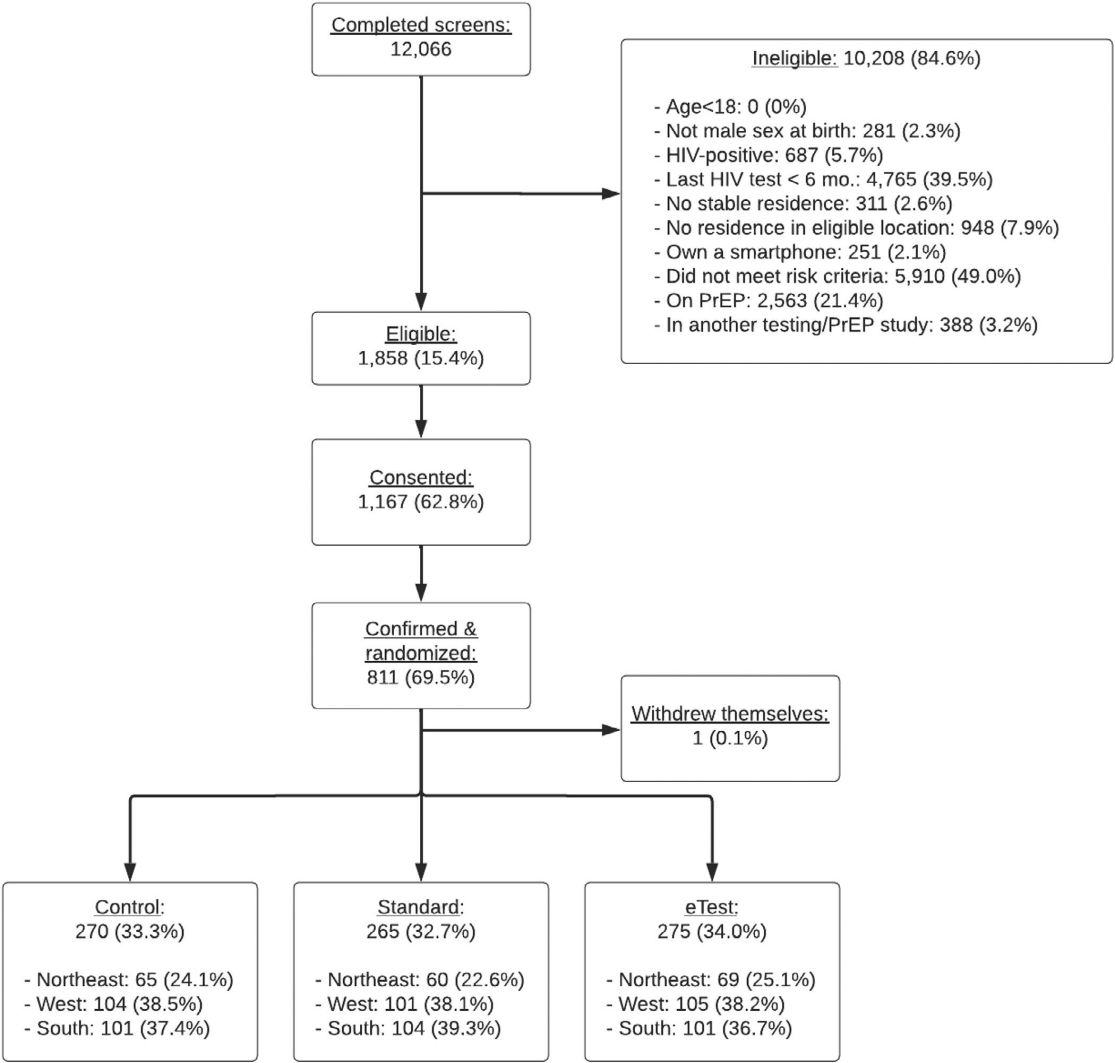
CONSORT diagram of screening and enrolment flow. PrEP, pre‐exposure prophylaxis.

Figure 1 shows a flow diagram of participants through recruitment milestones [16]. All participants were asked to complete online follow‐up surveys at 1, 4, 7, 10 and 12 months. Follow‐up surveys assessed HIV testing and other prevention and sexual health services received (e.g. STI testing, PrEP) since the previous survey, including both clinic‐based and home‐based services, as well as the results of any HIVST they used.

In all conditions, an email alert to counsellors was triggered each time a participant reported reactive or invalid test results from any HIV test. To link participants to care, counsellors contacted local HIV care clinics and assisted participants in scheduling an appointment via three‐way calling. For invalid results, participants were either referred to clinics or sent another HIVST for re‐testing according to their preference. At 12 months, we requested that all participants receive HIV testing from either a local clinic or via study‐provided HIVST according to their preferences, regardless of condition, and report their results. We requested that a portion of participants (*N* = 201) send photographic verification of these results. Otherwise, we did not explicitly instruct participants to use HIVST. Participants were compensated up to $250 based on their completion of follow‐up surveys.

Those assigned to the standard condition received HIVST kits with standard follow‐up resources, which included OraSure's free hotline. HIVST kits sent to participants in the eTest condition were equipped with Bluetooth sensors that detected when kits were opened and triggered an email alert to study counsellors to follow up with participants. Post‐test counselling was modelled after standard HIV test counselling based on the RESPECT‐2 model [17, 18]. Counsellors also provided basic PrEP education, which included providing information about its purpose, efficacy, side effects and how participants could access it. All counsellors were licensed HIV test counsellors in Rhode Island. A postcard was included in all HIVST kits with local HIV prevention, sexual health, general healthcare, mental health and alcohol/drug treatment resources. All study materials were available in both English and Spanish and were provided in participants’ primary language preferences.

### 2.4 Outcomes

#### 2.4.1 HIV testing

Surveys asked participants to report the number of times they had been tested for HIV since their previous survey, and the date, location (i.e. at a clinic, at home) and results of each test. Participants who reported testing via HIVST were asked whether the test was study‐provided. Those who reported testing at a clinic were asked to sign a release that allowed staff to verify these responses [16].

#### 2.4.2 Receipt of other prevention services

Surveys also asked participants whether they had received STI testing, sought a prescription for PrEP or were prescribed PrEP since their previous survey. Those who reported receiving these services were asked to report the dates, and results of these services, and to sign a release that would enable staff to verify these responses.

#### 2.4.3 CAS events

Participants were also asked to complete an online Timeline Followback [19] of their sexual behaviour over the past 30 days [20]. For each sex event, participants were asked to report details of each event (such as partner characteristics, specific acts). To capture the total number of “high risk” CAS events in a given month, we calculated the total number of times participants reported engaging in receptive or insertive anal sex without using a condom with a partner they were not sexually exclusive with, or who had HIV but were either not on treatment or their treatment status was unknown.

### 2.5 Statistical analysis

A priori power analysis suggested that the sample size needed to detect small effects across conditions when α = 0.05 and power = 0.8 was 720 [16]. Participants were considered lost to follow‐up if they did not complete three or more consecutive follow‐up surveys. Primary outcomes were receipt of (1) any HIV test, and (2) repeat HIV testing (> 1 test) over the course of the 12‐month study period, as well as (3) HIV testing in a given follow‐up period. Secondary outcomes were (1) receipt of a PrEP prescription, (2) STI testing during the study period and (3) the total number of high‐risk CAS events in a given month. We used logistic regression with dummy‐coded condition assignment as a predictor to test differences in outcomes across experimental conditions. A dummy‐coded covariate indicating whether participants reported testing fewer than three times in the 3 years prior to enrolling was included in all models of HIV testing. A similar covariate for STI testing was included in the STI testing model, and a binary variable reflecting whether participants had ever had a PrEP prescription in the past was included for the PrEP prescription model. We specified two‐way interactions between these covariates and condition assignment in all models, but none were significant and were excluded.

We fit longitudinal mixed effects models for two outcomes, HIV testing and high‐risk CAS events within a given follow‐up period, given that these outcomes varied within participants across the study period. We specified distributions appropriate for each outcome (logistic for HIV testing and negative binomial for high‐risk CAS events) with suitable link functions, unstructured covariance structures and robust standard errors. Time was included as a continuous covariate. A covariate reflecting pre‐enrolment HIV testing and baseline CAS events were included in these models. We used an intent‐to‐treat approach for all analyses. Missing data were considered missing at random. To provide conservative estimates in overall logistic models, we assumed that those with missing data did not engage in a given outcome (e.g. no testing, no PrEP) in a specified time period. Analyses were performed in Stata SE 16 (College Station, TX, USA).

## 3 RESULTS

See Figure 1 for enrolment flow. Although 1167 registered for the study, 811 participants were confirmed and randomized to condition. One participant explicitly withdrew. Table 1 shows demographic characteristics by condition assignment. Groups were generally well‐balanced with respect to all factors, except that fewer participants assigned to eTest reported ever having had a PrEP prescription. A higher percentage of those assigned to the control condition were lost to follow‐up prior to 12 months. Overall, 25% of participants stopped responding before 12 months. Fifty percent completed all five follow‐up surveys. Ninety‐seven percent of participants completed follow‐up surveys at month 1, but this rate declined across the follow‐up period: 78% completed month 4, 67% completed month 7, 62% completed month 10 and 64% completed month 12.

**Table 1:** Demographic and behavioural characteristics of the analysed sample (*N* = 810)

Characteristics	All (*N* = 810)	Control (*N* = 270)	Standard (*N* = 265)	eTest (*N* = 275)
	Mean (*SD*) or *N* (%)	Mean (*SD*) or *N* (%)	Mean (*SD*) or *N* (%)	Mean (*SD*) or *N* (%)
Age (Range: 18–81, *M* ± SD)	34.7 (12.1)	34.5 (11.4)	34.6 (12.6)	35.2 (12.3)
Trans/other gender identity	15 (1.9)	1 (0.4)	7 (2.6)	7 (2.5)
Race				
White	520 (64.2)	179 (66.3)	169 (63.8)	172 (62.5)
Black or African American	85 (10.5)	21 (7.8)	33 (12.5)	31 (11.3)
American Indian/Alaska Native	7 (0.9)	2 (0.7)	2 (0.8)	3 (1.1)
Asian	72 (8.9)	25 (9.3)	24 (9.1)	23 (8.4)
Pacific Islander/Native Hawaiian	2 (0.2)	0 (0.0)	1 (0.4)	1 (0.4)
Multiracial	59 (7.3)	20 (7.4)	16 (6.0)	23 (8.4)
Chose not to respond	65 (8.0)	23 (8.5)	20 (7.5)	22 (8.0)
Ethnicity (Hispanic or Latino)	277 (34.2)	95 (35.2)	84 (31.7)	98 (35.6)
Spanish primary language	56 (6.9)	16 (5.9)	16 (6.0)	24 (8.7)
Single relationship status	437 (54.0)	132 (48.9)	144 (54.3)	161 (58.6)
College degree	420 (51.9)	137 (50.7)	144 (54.3)	139 (50.6)
Low income^a^	243 (30.0)	71 (26.3)	80 (30.2)	92 (33.5)
Unemployed	130 (16.1)	45 (16.7)	39 (14.7)	46 (16.7)
Gay or bisexual identity	774 (95.6)	258 (95.6)	254 (95.9)	262 (95.2)
Region				
Northeast	194 (24.0)	65 (24.1)	60 (22.6)	69 (25.1)
South	306 (37.8)	101 (37.4)	104 (39.3)	101 (36.7)
West	310 (38.3)	104 (38.5)	101 (38.1)	105 (38.2)
Never tested for HIV, lifetime	97 (12.0)	35 (13.0)	28 (10.6)	34 (12.4)
Last HIV test, in years	2.9 (5.1)	2.7 (4.9)	2.9 (5.4)	3.0 (5.0)
<1 HIV test/year, past 3 years	486 (60.0)	157 (58.2)	163 (61.5)	166 (60.4)
< 1 STI test/year, past 3 years	611 (75.4)	205 (75.9)	197 (74.3)	209 (76.0)
High‐risk CAS events, past 30 days	1.3 (2.6)	1.0 (1.8)	1.3 (2.3)	1.4 (3.4)
CAS with high‐risk partner^b^	718 (88.6)	239 (88.5)	229 (86.4)	250 (90.9
Regular sex w/ HIV‐positive partner^b^	84 (10.4)	32 (11.9)	29 (10.9)	23 (8.4)
Diagnosed w/ STI	90 (11.1)	26 (9.6)	30 (11.3)	34 (12.4)
Ever had PrEP prescription	112 (13.8)	45 (16.7)	40 (15.1)	27 (9.8)
Lost to follow‐up^c^	197 (24.3)	79 (29.3)	53 (20.0)	65 (23.6)
Average % follow‐up surveys completed	73.6 (31.4)	69.5 (32.5)	76.3 (30.4)	75.0 (30.8)

*Note*: All questions referred to the past 12 months.

Abbreviations: CAS, condomless anal sex; STI, sexually transmitted infection.

^a^ Represents those with a household income <$30,000 a year.

^b^ Participants must have met one or more of these criteria for PrEP eligibility to enrol.

^c^ Represents participants who did not complete three or more consecutive follow‐up surveys.

### 3.1 eTest app use and HIVST kit detection

eTest participants reported having kept the app on their phones for an average of 11.5 months (*SD* = 2.0, Range = 1−12) during the study period. The eTest system detected a kit opening during 62.3% of follow‐up periods in which participants reported using a study‐provided HIVST. Participants were ultimately reached by phone and provided counselling for 95.1% of these kit‐opening alerts. Sixty‐nine percent of all eTest participants were provided with counselling at least once during the study.

### 3.2 Reactive and invalid HIVST results

**Figure 2:**
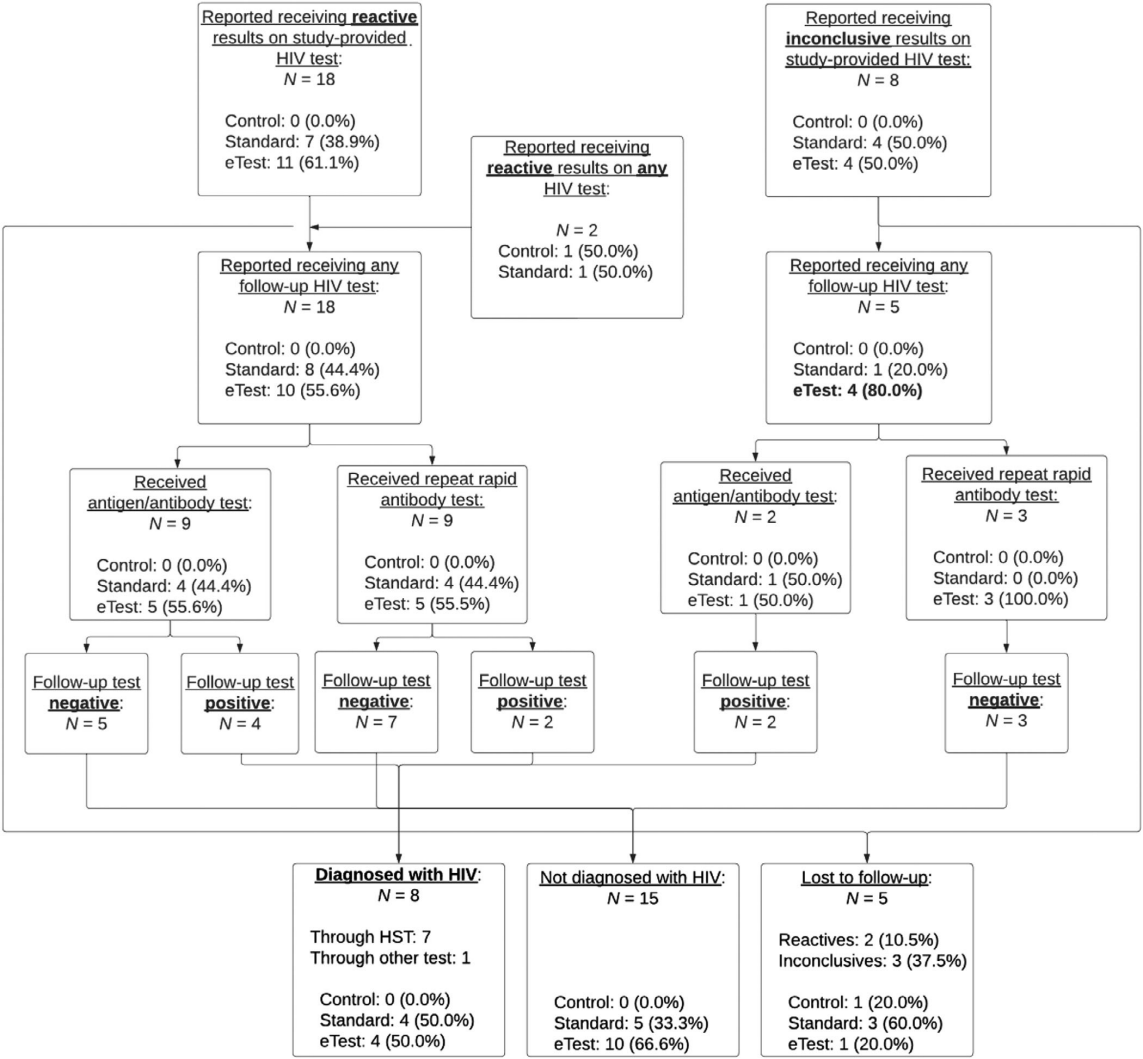
Flow of all reported reactive and invalid test results throughout the study period.

Figure 2 shows a flow of all reported reactive and invalid HIV testing results, both through study‐provided HIVST and non‐study testing. A total of 2064 HIVST kits were sent to participants in the standard and eTest conditions. Twenty‐eight reactive or invalid tests were reported during the study, eight of which led to an HIV diagnosis (28.6%). Follow‐up testing was negative for 15 of these reports (53.6%) and five (17.9%) were lost to follow‐up (37.5% of inconclusive vs. 10.5% of reactive). All but one new HIV diagnosis (87.5%) was identified originally through an HIVST.

### 3.3 HIV testing

**Figure 3:**
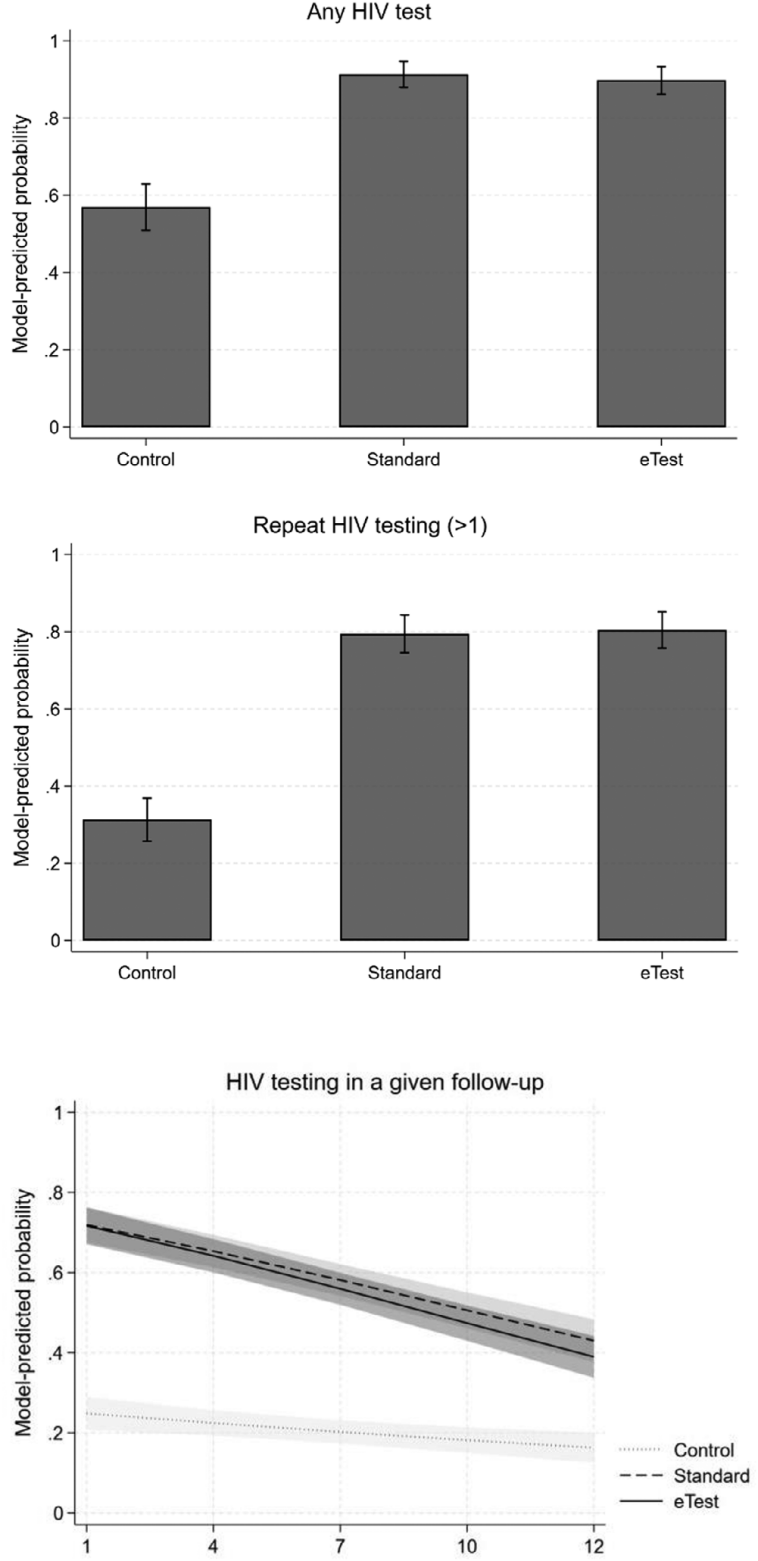
Model‐predicted probabilities of any (top panel) and repeat HIV testing (middle panel) and predicted effects on testing in a given follow‐up (bottom panel) over the 12‐month study period.

Table 2 shows the results of logistic and mixed regression models for all HIV testing outcomes. Relative to control group participants, the odds of completing any HIV test were 7.91 times higher among those in the standard group (95% CI = 4.9, 12.9) and 6.63 times higher among those in the eTest group (95% CI = 4.2, 10.5; see Table). Model‐adjusted probabilities of any testing were 91.0% in standard (95% CI = 87.9, 94.6), 89.4% in the eTest group (95% CI = 86.2, 93.3) and 57.0% in control (95% CI = 51.0, 62.9; see Figure 3 and Table 4). Pairwise contrasts between standard and eTest participants were not significant. In the model of repeat testing (>1 test over 12 months), the odds of reporting more than one test across the study period was 9.03 times higher in the eTest group (95% CI = 6.1, 13.4) and 8.47 times higher in the standard group (95% CI = 5.7, 12.6) compared to the control group. Model‐adjusted probabilities of repeat testing were 79.4% in standard (95% CI = 74.5, 84.3), 80.4% in eTest (95% CI = 75.7, 85.1) and 31.3% in control (95% CI = 25.7, 36.9). Finally, in a logistic mixed model of HIV testing across follow‐up periods, two‐way interactions between each condition and time were statistically significant. Although the model‐adjusted probabilities of reporting HIV testing in a given follow‐up period were significantly higher among both standard (57.8%, 95% CI = 54.0, 61.6) and eTest participants (56.2%, 95% CI = 51.8, 59.4) versus controls (22.0%, 95% CI = 17.6, 23.2), the probabilities of testing declined in all groups across the study period and declined more so among eTest (8.0%, 95% CI = −9.6, −6.4) and standard (7.2%, 95% CI = −8.7, −5.6) participants compared to controls (1.7%, 95% CI = −3.5, −0.8).

**Table 2:** Logistic regression and mixed models of HIV testing outcomes over the 12‐month study period

	Any HIV testing				Repeat (>1) HIV testing				HIV testing in a given F/U			
*Variable*	*OR*	*SE*	*p*	95% CI	*OR*	*SE*	*p*	95% CI	*OR*	*SE*	*p*	95% CI
< 1 test/year, past 3 years^a^	0.56	0.11	0.004	0.38−0.83	0.55	0.10	0.001	0.39−0.78	0.72	0.07	0.001	0.59−0.87
Standard condition	7.91	1.97	<0.001	4.85−12.88	8.47	1.72	<0.001	5.69−12.61	9.33	1.87	<0.001	6.29−13.83
eTest condition	6.63	1.55	<0.001	4.19−10.48	9.03	1.83	<0.001	6.07−13.44	9.59	1.97	<0.001	6.41−14.33
Time	–	–	–	–	–	–	–	–	0.88	0.04	0.003	0.80−0.96
Standard*time	–	–	–	–	–	–	–	–	0.84	0.05	0.003	0.75−0.94
eTest*time	–	–	–	–	–	–	–	–	0.81	0.05	<0.001	0.72−0.91

^a^ Dummy variable representing whether participants reported testing at least three times in the 3 years prior to enrolling in the study.

### 3.4 STI testing, PrEP uptake and sexual risk behaviour

**Figure 4:**
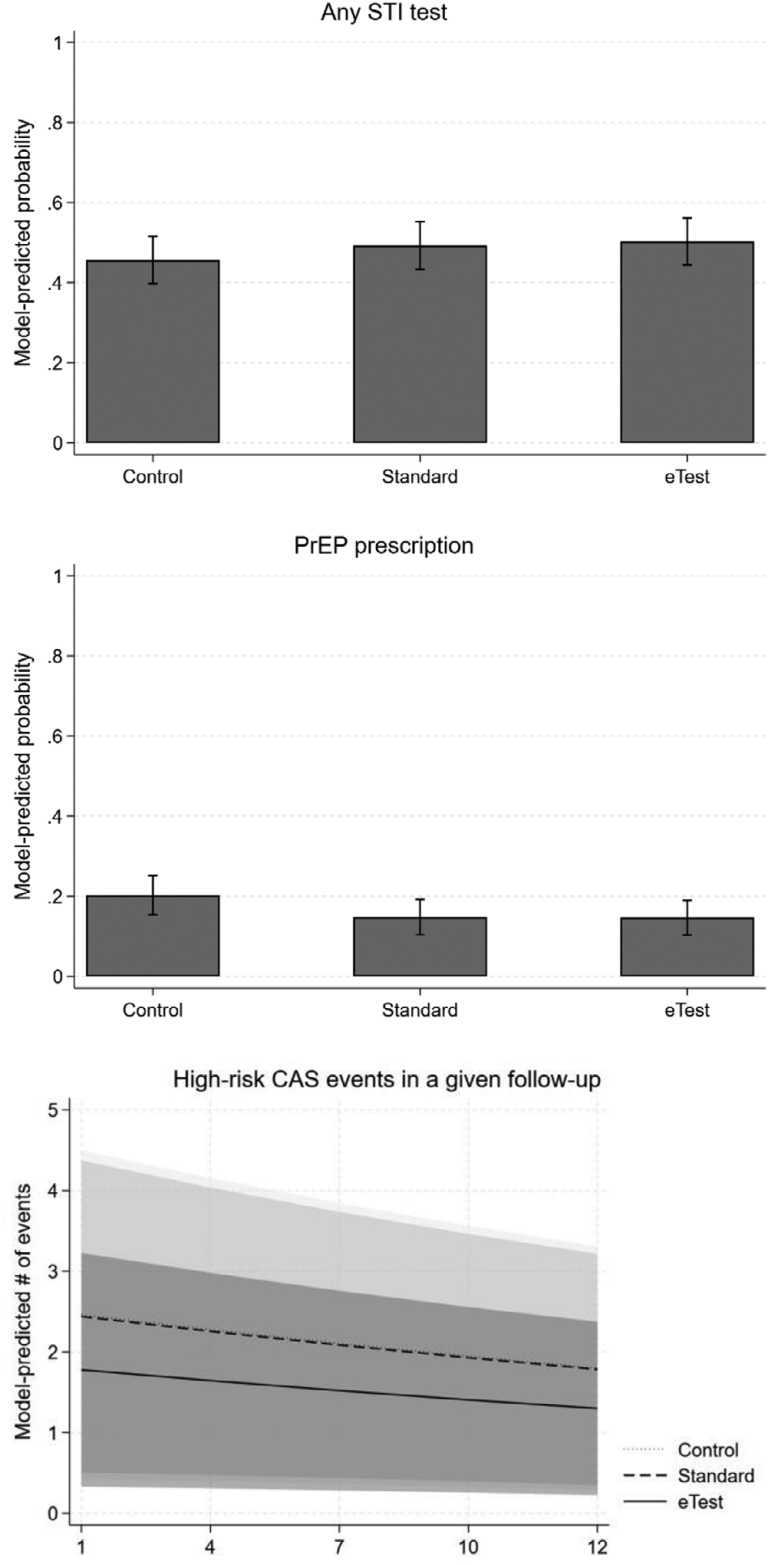
Model‐predicted probabilities of STI testing (top panel), PrEP uptake (middle panel) and sexual risk behaviour (bottom panel) over the 12‐month study period. CAS, condomless anal sex; PrEP, pre‐exposure prophylaxis; STI, sexually transmitted infection.

Table 3 shows logistic and mixed regression models for secondary outcomes: STI testing, PrEP uptake and sexual risk behaviour. Main effects comparing the standard and eTest conditions to controls for STI testing were not statistically significant, nor was a pairwise comparison testing differences between the eTest and standard condition (see Figure 4). Similarly, in a logistic model of PrEP uptake, none of the main effects or pairwise comparisons across groups were statistically significant. Finally, in a negative binomial mixed model of high‐risk CAS events in a given follow‐up period, a two‐way interaction between condition assignment and time was not significant and was excluded from the model. However, a main effect of time was significant, suggesting that the incidence rate of high‐risk CAS events decreased across all participants over each successive follow‐up period. A main effect of eTest condition assignment was also significant, suggesting that the rate of high‐risk CAS events was lower among these participants relative to control participants across all time points. Contrasts suggested that eTest participants reported fewer high‐risk CAS events relative to standard group participants (*IRR* = 0.73, *SE* = 0.12, 95% CI = 0.53−0.99). Across all time points, the average predicted number of high‐risk CAS events was 1.46 in the eTest group (95% CI = 0.3, 2.6), versus 2.01 in the standard group (95% CI = 0.5, 3.5) and 2.03 in the control group (95% CI = 0.5, 3.6).

**Table 3:** Logistic regression and mixed models of secondary outcomes over the 12‐month study period

*Variable*	STI testing				PrEP prescription				High‐risk CAS events			
	*OR*	*SE*	*p*	95% CI	*OR*	*SE*	*p*	95% CI	*IRR*	*SE*	*p*	95% CI
< 1 test/year, past 3 years^a^	0.50	0.08	<0.001	0.36−0.69	1.18	0.05	<0.001	1.08−1.29	–	–	–	–
Ever had PrEP Rx^b^	–	–	–	–	2.16	0.52	0.001	1.35−3.45	–	–	–	–
High‐risk CAS @ BL^c^	–	–	–	–	–	–	–	–	1.31	0.04	<0.001	1.24−1.38
Time	–	–	–	–	–	–	–	–	0.92	0.02	0.003	0.88−0.97
Standard condition	1.16	0.20	.398	0.82−1.63	0.67	0.16	.103	0.43−1.08	0.99	0.16	.956	0.73−1.35
eTest condition	1.21	0.21	.273	0.86−1.70	0.67	0.16	.094	0.42−1.07	0.72	0.12	.047	0.52−0.99

^a^ Dummy variable representing whether participants reported testing for sexually transmitted infections (STIs) at least three times in the 3 years prior to enrolling in the study.

^b^ Dummy variable representing whether participants reported ever having an HIV pre‐exposure prophylaxis (PrEP) prescription before in their lifetimes.

^c^ Variable representing the number of high‐risk condomless anal sex (CAS) events participants reported in the 30 days before enrolling in the study.

**Table 4:** Model‐predicted probabilities for each study outcome by condition assignment over the 12‐month study period

	Control		Standard		eTest	
	*N*	%	*N*	%	*N*	%
Diagnosed with HIV	0	0.0	4	1.5	4	1.5

	Estimate	95% CI	Estimate	95% CI	Estimate	95% CI
Any HIV test	0.57	0.51−0.63	0.91	0.88−0.95	0.89	0.86−0.93
Repeat testing (>1 test)	0.31	0.26−0.37	0.79	0.75−0.84	0.80	0.76−0.85
HIV testing in a given follow‐up period	0.22	0.18−0.23	0.58	0.54−0.62	0.56	0.52−0.59
STI testing	0.46	0.40−0.52	0.49	0.43−0.55	0.50	0.44−0.56
PrEP uptake	0.20	0.15−0.25	0.15	0.10−0.19	0.15	0.10−0.19
High‐risk CAS events^a^	2.03	0.45−3.61	2.01	0.51−3.50	1.46	0.34−2.59

Abbreviations: CAS, condomless anal sex; PrEP, pre‐exposure prophylaxis; STI, sexually transmitted infection.

^a^ Reflects model‐predicted number of high‐risk CAS events in the past 30 days.

## 4 DISCUSSION

Findings from this study provide several important insights about the role of HIVST in efforts to diagnose more new cases earlier in the United States, as well as optimal ways of providing follow‐up alongside HIVST. First, our results showed that about 1% of study participants were ultimately diagnosed with HIV during the study period, and all but one of these were diagnosed through HIVST. These findings suggest that regular home delivery of HIVST is a key strategy that could help diagnose new HIV cases earlier, especially among those who are at increased risk but who test infrequently. However, of those who reported receiving reactive results on a study‐provided HIVST, over 70% reported receiving negative results on follow‐up testing. Although it is possible that some of these reports may have reflected misread or misinterpreted results, it is unlikely that these errors alone would account for such a sizable number of reports. For comparison, of the 201 photos of validated HIVSTs we collected at 12 months, only about 3% had evidence that participants may have misinterpreted or misreported their results. Nevertheless, although a substantial number of reported reactive results were negative, the overall rate of such results that were falsely reactive is likely to be low (0.5−0.9%).

Our results also showed that regularly delivering HIVST directly to MSM who are at high risk but who test irregularly substantially increased rates of HIV testing and encouraged more regular testing. The odds that a participant tested for HIV over the 12‐month period if they were sent HIVSTs quarterly was 7−8 times that of those who were simply reminded to get tested at a local clinic. Likewise, the odds that a participant tested more than once in a year with HIVST was 8−9 times higher with HIVST than with clinic‐based testing. These findings contrast with past studies, which have shown smaller differences in test completion when comparing HIVST versus clinic‐based testing (RR = 1.45) [7]. These findings also extend previous studies by showing that although HIVST promotes impressive increases in testing initially, these gains may decrease over time. The probability of testing for HIV in any given follow‐up period declined by 7−8% across the study period among those receiving HIVST.

One of the most novel contributions of this trial was that it tested the benefits of one approach to providing support alongside HIVST: Providing timely follow‐up post‐test counselling over the phone. However, our findings did not provide strong evidence that monitoring HIVST use and providing follow‐up phone counselling and basic PrEP education increased rates of engagement with other prevention services, such as STI testing and PrEP uptake. These results provide additional evidence to help reconcile mixed findings in past studies [9, 10, 15], suggesting that those who use HIVST seek STI testing as often as those referred for clinic‐based testing. However, our findings also extend these results to PrEP uptake, showing that although those who used HIVST were not *less* likely to seek or start PrEP than those referred for clinic‐based testing, providing timely post‐test counselling and basic PrEP education was not effective in increasing rates of PrEP uptake. This pattern of findings suggests that, although HIVST could serve as an opportune time to encourage PrEP use, to effectively increase uptake, interventions are needed that explicitly address key determinants of PrEP uptake.

Notably, in contrast with past studies of clinic‐based HIV testing showing that post‐test counselling did not reduce risk behaviour and STI diagnoses [17], our findings suggest that providing risk reduction counselling alongside HIVST could reduce risk behaviour, given that eTest participants reported an average of 27−28% fewer high‐risk CAS events at a given follow‐up relative to control and standard group participants after controlling for baseline CAS events. However, there was also considerable variability in this outcome within conditions, such that pairwise comparisons only narrowly met the criteria for statistical significance (*p*<.05). These findings also rely exclusively on self‐report. As such, while phone‐based post‐test counselling could help reduce self‐reported HIV risk behaviour, it may not be sufficient enough to justify the considerable expense of providing monitoring (e.g. equipment and software maintenance and updates, counselling).

Although this trial has important strengths, several limitations are important to note. First, since COVID‐19 stay‐at‐home orders were in effect in most states for roughly 6 months of our 4‐year study period, it is possible that these orders encouraged more use of HIVST than past studies not affected by COVID‐19. However, our data do not provide compelling evidence that HIVST use was either statistically or meaningfully different between 15 March and 31 August 2020, when most stay‐at‐home orders were in effect, suggesting that this is unlikely to explain HIVST's much larger effects in our study compared to past studies. Second, the outcomes reported in this manuscript were largely based on self‐report, meaning that some findings may be less accurate compared to relying on biomarkers or other non‐self‐report outcomes. However, the kit tracking approach we used in this study provides some validation of when kits were indeed used. Participants were also informed that we would verify any reports of clinical services and were asked to authorize these providers/clinics to release their information to us. Third, although the percentage of participants who identified as a racial or ethnic minority was relatively high overall (54.9%), the percentage of Black/African American participants was slightly below the general population and far below what is needed, given their importance to HIV prevention. Dedicated research is needed to identify effective ways to engage Black/African American MSM in HIV research and programming, both online and in‐person.

## 5 CONCLUSIONS

In summary, we found that HIVST vastly increased HIV testing and encouraged more regular testing among MSM who were at high risk for HIV but who had previously not tested often. HIVST also identified nearly all new cases in this study, suggesting that it could play a critical role in diagnosing HIV earlier. Providing timely follow‐up counselling after HIVST did not increase rates of STI testing or PrEP uptake, although some evidence suggested that counselling may have reduced sexual risk behaviour. Future research should explore other ways of providing support alongside HIVST, implementation strategies that might promote more use of HIVST and ways of optimizing the delivery of HIVST to maximize uptake [21].

## COMPETING INTERESTS

We declare no competing interests.

## AUTHORS’ CONTRIBUTIONS

TBW conceptualized the research and led data collection, data analysis and manuscript preparation. PAC conceptualized the research, assisted in securing funding, helped lead data collection and prepared drafts of this manuscript. JDK conceptualized the research, assisted in security funding, helped lead data collection and assisted with manuscript preparation. LMW helped lead data collection and assisted with manuscript preparation. EMSO served as project director and supervised all research staff and study counsellors, oversaw all data collection activities, helped with data management and assisted in preparing this manuscript. DJC led all recruitment activities, assisted with data collection and helped prepare drafts of this manuscript. JPG helped supervise data collection and helped prepare and revise drafts of the manuscript. SN served as a study counsellor, assisted with data collection, helped with data management and assisted in preparing this manuscript.

## FUNDING

Funding for this work was provided by the National Institute of Mental Health grant R01MH114891.

## Supporting information


**Appendix A**: Full list of eTest study cities/states

## Data Availability

Deidentified data that support the findings of this study are available from the corresponding author upon reasonable request.
