# Supplementary material for: The effects of regular home delivery of HIV self‐testing and follow‐up counselling on HIV testing and prevention outcomes in men who have sex with men who test infrequently in the United States: a pragmatic, virtual randomized controlled trial
Source: J Int AIDS Soc. 2024 Jul 17;27(7):e26318. doi: 10.1002/jia2.26318 (PMC11254576; doi:10.1002/jia2.26318)
Supplement: Supplementary file 1 — Appendix A: Full list of eTest study cities/states [file JIA2-27-e26318-s001.docx]

Appendix A

Full list of eTest study cities/states

Northeast:

- Boston, Massachusetts
- Providence, Rhode Island

South:

- Baton Rouge, Louisiana
- Jacksonville, Florida
- Miami, Florida
- Mississippi
- New Orleans, Louisiana
- Orlando, Florida
- Shreveport, Louisiana
- Tallahassee, Florida

West:

- Los Angeles, California
